# Discovery and Selection of Hepatitis B Virus-Derived T Cell Epitopes for Global Immunotherapy Based on Viral Indispensability, Conservation, and HLA-Binding Strength

**DOI:** 10.1128/JVI.01663-19

**Published:** 2020-03-17

**Authors:** Monique T. A. de Beijer, Diahann T. S. L. Jansen, Yingying Dou, Wim J. E. van Esch, Juk Yee Mok, Mariëlle J. P. Maas, Giso Brasser, Robert A. de Man, Andrea M. Woltman, Sonja I. Buschow

**Affiliations:** a Department of Gastroenterology and Hepatology, Erasmus MC University Medical Center Rotterdam, Rotterdam, the Netherlands; b Sanquin, Amsterdam, the Netherlands; University of Southern California

**Keywords:** hepatitis B virus, HBx, polymerase, cytotoxic T cells, epitope selection, epitope discovery, immunotherapy

## Abstract

Immunotherapy represents an attractive option for the treatment of chronic hepatitis B virus (HBV) infection. The HBV proteins polymerase (Pol) and HBx are of special interest for antigen-specific immunotherapy because they are essential for viral replication and have been associated with viral control (Pol) or are still expressed upon viral DNA integration (HBx). Here, we scored all currently described HBx- and Pol-derived epitope sequences for viral indispensability and conservation across all HBV genotypes. This yielded 7 HBx-derived and 26 Pol-derived reported epitopes with functional association and high conservation. We subsequently predicted novel HLA-binding peptides for 6 HLA supertypes prevalent in HBV-infected patients. Potential epitopes expected to be the least prone to immune escape were subjected to a state-of-the-art *in vitro* assay to validate their HLA-binding capacity. Using this method, a total of 13 HLA binders derived from HBx and 33 binders from Pol were identified across HLA types. Subsequently, we demonstrated interferon gamma (IFN-γ) production in response to 5 of the novel HBx-derived binders and 17 of the novel Pol-derived binders. In addition, we validated several infrequently described epitopes. Collectively, these results specify a set of highly potent T cell epitopes that represent a valuable resource for future HBV immunotherapy design.

**IMPORTANCE** Multiple HBV-derived T cell epitopes have been reported, which can be useful in a therapeutic vaccination strategy. However, these epitopes are largely restricted to HLA-A*02, which is not dominantly expressed in populations with high HBV prevalence. Thus, current epitopes are falling short in the development of a global immunotherapeutic approach. Therefore, we aimed to identify novel epitopes for 6 HLA supertypes most prevalent in the infected population. Moreover, established epitopes might not all be equally effective as they can be subject to different levels of immune escape. It is therefore important to identify targets that are crucial in viral replication and conserved in the majority of the infected population. Here, we applied a stringent selection procedure to compose a combined overview of existing and novel HBV-derived T cell epitopes most promising for viral eradication. This set of T cell epitopes now lays the basis for the development of globally effective HBV antigen-specific immunotherapies.

## Abstract (precis)

Multiple HBV-derived T cell epitopes have been reported, which can be useful in a therapeutic vaccination strategy. However, these epitopes are largely restricted to HLA-A*02, which is not dominantly expressed in populations with high HBV prevalence. Thus, current epitopes are falling short in the development of a global immunotherapeutic approach. Therefore, we aimed to identify novel epitopes for 6 HLA supertypes most prevalent in the infected population. Moreover, established epitopes might not all be equally effective as they can be subject to different levels of immune escape. It is therefore important to identify targets that are crucial in viral replication and conserved in the majority of the infected population. Here, we applied a stringent selection procedure to compose a combined overview of existing and novel HBV-derived T cell epitopes most promising for viral eradication. This set of T cell epitopes now lays the basis for the development of globally effective HBV antigen-specific immunotherapies.

## INTRODUCTION

Chronic hepatitis B virus (CHB) infection affects roughly 250 million people worldwide (1) and is a main cause of cirrhosis and hepatocellular carcinoma (HCC). Chronically infected patients can be treated with expensive viral replication inhibitors, but complete viral eradication as in hepatitis C virus infection is rare. In fact, curative treatment remains highly demanded since CHB infection is expected to remain a global health problem for many years (2).

Immunotherapy had already emerged in the 1990s as a promising option to treat CHB. T cell responses are considered essential for viral clearance but are scarce or exhausted in CHB patients (3–7). Still, they can be boosted or induced via several therapeutic strategies, e.g., vaccination or adoptive transfer of engineered hepatitis B virus (HBV)-specific T cells (8–22). However, these strategies are hampered by the lack of an HLA-broad epitope repertoire against which antiviral T cells can be directed. The majority of currently described epitopes are restricted to HLA-A*02, which is highly prevalent in Caucasians (23, 24). Yet HLA-A*02 is expressed in only roughly 40% of the world’s population and is not dominant in Asian and African populations (23), whereas especially these populations show a high HBV prevalence (2). Thus, it is vital to identify non-HLA-A*02-restricted epitopes, especially for HLA types prevalent among Asians and Africans, such as HLA-A*24 or HLA-B*07.

Next to a lack of HLA diversity, the current HBV-derived epitope repertoire is skewed by the fact that many reports focus on dissecting T cell responses against the HBV surface antigen (HBsAg) or core antigen (HBcAg). However, the proteins X (HBx) and polymerase (Pol) also pose interesting targets, as both are vital for viral persistence (25–27) and interfere with the antiviral immunity of the host (28). Furthermore, HBx is expressed only in infected hepatocytes and is involved in the development of HCC (29). The expression of HBx is likely to be retained upon HCC formation because of the productive integration of the HBx gene into the host genome (30–32). Collectively, this provides a rationale to target HBx in patients suffering from CHB as well as HBV-related HCC (33, 34). Besides HBx, Pol represents an interesting immunotherapeutic target. Pol is more immunogenic than HBsAg in HBV transgenic mice (35), and high frequencies of Pol-specific T cells are associated with viral control after discontinuation of viral replication inhibitors in patients (36). This implies that Pol-specific T cells retain their function throughout the course of chronicity and can contribute to immune control *in vivo*. Others have explored strategies to predict HLA-I epitopes from Pol but focused exclusively on a single HLA type or assessed only a limited number of HBV sequences (37–40). Taken together, the identification of novel, non-HLA-A*02-restricted HLA-I epitopes derived from HBx and Pol would greatly benefit generic anti-HBV immunotherapy design.

In addition to a limited epitope repertoire, there is another hurdle in the development of HBV-directed immunotherapy. Established epitopes might not all be equally effective, as they can be subjected to different levels of viral mutagenesis and subsequent immune escape. Indeed, previous reports clearly demonstrate that HBV is subject to immune pressure and that mutation of epitope sequences leads to immune evasion (41–43) or even HBV reactivation (44, 45). Importantly, in the case of immune escape, responsive memory T cells may still linger in patients despite the loss of epitope presentation on target cells. Thus, prevalent detection of cognate T cells by itself offers no guarantee of clinical relevance. Long-term efficacy of generic immunotherapy can be expected to be dictated by epitope preservation across the patient population, in which infection is caused by different HBV genotypes. Moreover, amino acids conserved between genotypes are more likely to have functional importance to the virus. After all, mutation of functional sequences would lead to a loss of viral fitness, which drives subsequent negative selection. Indeed, amino acids essential for HBV replication are almost exclusively highly conserved (46–48). Taken together, T cell responses directed against conserved epitopes from functional protein domains would benefit the majority of patients while simultaneously hampering viral replication and immune escape.

Here, we have taken an effort to tackle the above-mentioned issues by integrating viral indispensability, genomic variation, HLA binding, and immunogenicity to identify the best HBx- and Pol-derived T cell epitopes for immunotherapy across 6 of the most prevalent HLA supertypes within the HBV-infected population. The results of this study pave the way for the development of globally effective HBV antigen-specific immunotherapies.

## RESULTS

### Ranking of the most optimal reported HBx- and polymerase-derived epitopes.

**FIG 1:**
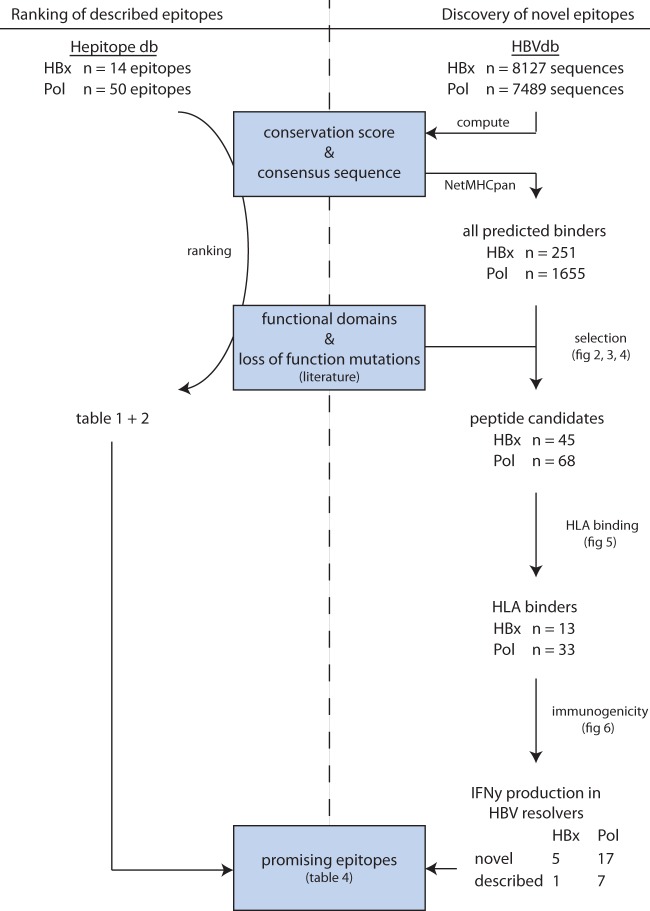
Workflow of epitope ranking and discovery. All amino acid sequences from HBx and Pol were extracted from the publicly available database HBVdb and used to compute a consensus sequence and conservation score (top right) (see Materials and Methods). The resulting consensus sequences were subsequently used in NetMHCpan to predict sequences of HLA-binding peptides across the 6 most common HLA supertypes in the HBV-infected population. This yielded 251 and 1,655 HBx- and Pol-derived potential HLA binders, respectively. A total of 113 of these were selected for *in vitro* validation based on predicted HLA binding, conservation, and reported functional association within the viral protein. This yielded 13 HBx-derived and 33 Pol-derived validated HLA binders. All of these were subsequently tested for immunogenicity, in which 6 HBx- and 24 Pol-derived peptides elicited IFN-γ responses in an ELISA. In addition, all currently known Pol- and HBx-derived epitopes were extracted from the publicly available database Hepitopes (top left) and ranked according to conservation score and reported functional association. These findings are summarized in Table 4 (bottom center).

**FIG 2:**
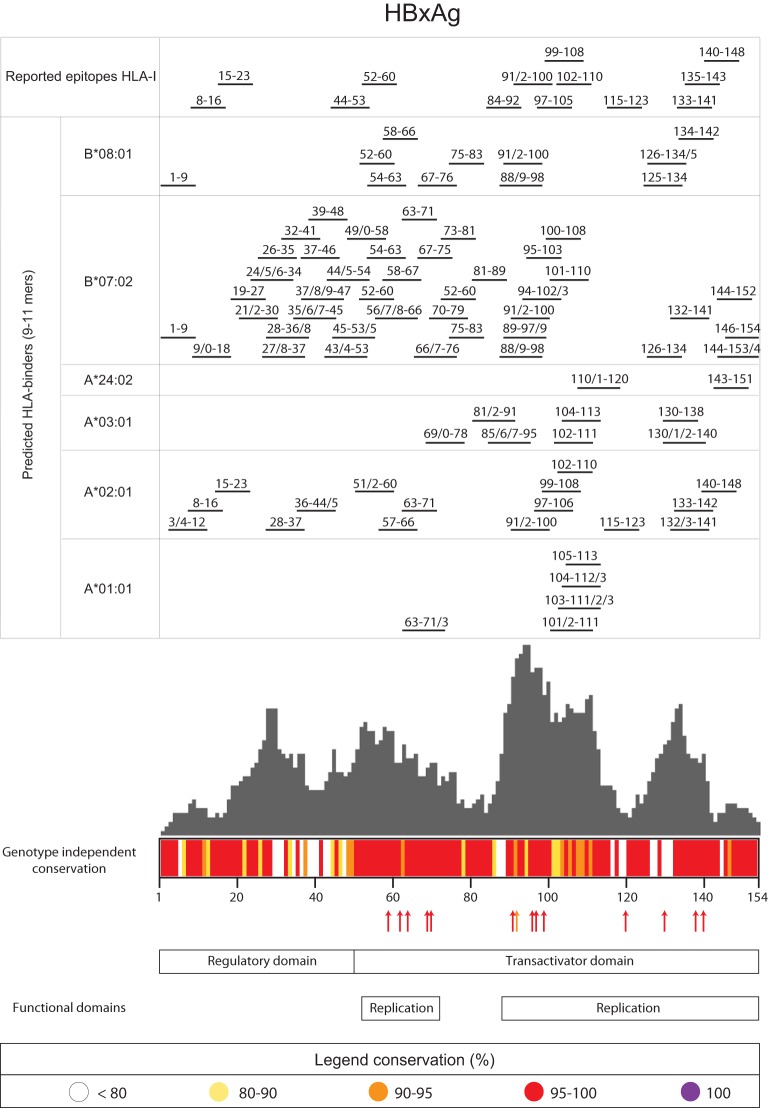
Alignment of reported and predicted CD8^+^ T cell targets based on protein conservation and function for HBx. The centered bar diagram depicts the length of the consensus sequence of the HBx protein (see Materials and Methods), in which the conservation score across viral genotypes is indicated by a color code (key) for each amino acid. Reported epitopes obtained from the Hepitopes database are aligned to this sequence and shown on top. Below this, potential novel binders predicted by NetMHCpan (9 to 11 amino acids) are depicted for each HLA supertype representative. The gray histogram represents the frequency of each amino acid within all predicted binders (8 to 14 amino acids long) over the protein sequence. Essential amino acids for which mutation leads to a loss of viral persistence are indicated by arrows matching the color of the conservation score. Functional domains are depicted at the bottom according to the nomenclature of HBVdb. References describing the experimental evidence for essential amino acids and functional domains are listed in Table S1 in the supplemental material.

**FIG 3:**
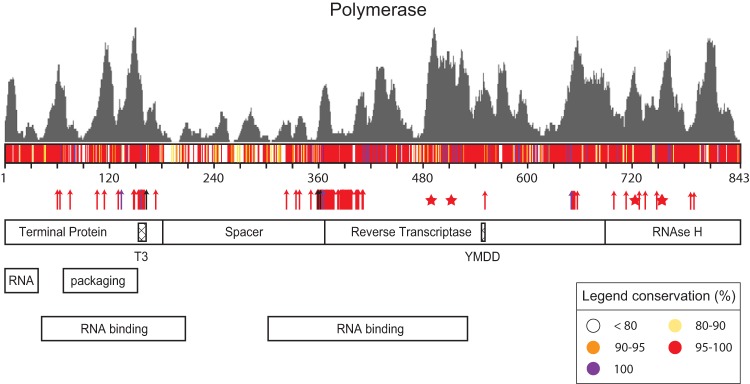
Alignment of reported and predicted CD8^+^ T cell targets based on protein conservation and function for polymerase. The gray histogram represents the frequency distribution of predicted binders (8 to 14 amino acids long) over the protein sequence. The conservation score (key) of each amino acid is shown as a horizontal color-coded bar diagram. Essential amino acids for which a single or combined mutation leads to a loss of viral persistence (≥50%) are indicated by arrows matching the color of the conservation score for that particular amino acid. Amino acids that are predicted to be vital for three-dimensional (3D) conformation are indicated with stars. General domains are depicted according to previously determined nomenclature (48), in which the T3 domain and the YMDD motif are also represented. References describing the experimental evidence for essential amino acids and functional domains are listed in Table S2 in the supplemental material. A high-resolution figure depicting described epitopes and potential novel binders (9 to 11 amino acids) is available in Fig. S2 in the supplemental material to allow zoom-in on single peptides and regions of interest.

We first set out to rank reported epitopes for HBx and Pol based on protein conservation and function using a comprehensive database called Hepitopes (24, 49). From Hepitopes, we extracted all unique epitopes identified in human hosts for HBx (*n* = 14) and Pol (*n* = 50) (Fig. 1, left), which were found to be largely HLA-A*02 restricted (see Fig. S1 in the supplemental material). To rank these epitopes based on conservation, all protein sequences for HBx and Pol were extracted from a large public HBV repository (HBVdb) (50, 51) (Fig. 1, right) and used to compute an overall consensus sequence across viral genotypes (see Materials and Methods). Based on this sequence, a conservation score (prevalence) was calculated (Fig. 2 and 3 for HBx and Pol, respectively). To additionally rank functional associations, we extended our analysis to described functional domains and amino acids that severely impaired viral function upon mutation (see Tables S1 and S2 in the supplemental material for details on reviews and studies used) (26, 46–48, 52–69). These were additionally aligned to the consensus sequences of HBx and Pol (Fig. 2 and 3, respectively, arrows and bottom panels). As expected, functional domains and essential amino acids mostly aligned to highly conserved regions. Finally, we used the acquired information on conservation and functionality to rank all reported HBx- and Pol-derived epitopes (Tables 1 and 2, respectively).

**TABLE 1:** Ranking of reported epitopes for HBx based on conservation and function^*a*^

Amino acid positions	Amino acid sequence	HLA type	Cons (%)	No. of papers reporting epitope	Presence of functional amino acid/domain	% responders (no. of responders/total no. of subjects tested)		
						Acute	Chronic	HCC
*133–141*	*VLGGCRHKL*	A*02:01	*98.1*	*2*	*Yes*/*yes*		*0* (*0*/*20*)	*0* (*0*/*10*)
*52–60*	*HLSLRGLPV*	A*02:01	*98.0*	*3*	*Yes*/*yes*		*0* (*0*/*20*)	*19* (*3*/*16*)
*135–143*	*GGCRHKLVC*	A*11:01	*97.7*	*1*	*Yes*/*yes*			
*92–100*	*VLHKRTLGL*	A*02:01	*89.8*	*5*	*Yes*/*yes*		*0* (*0*/*20*)	*19* (*3*/*16*)
*91–100*	*KVLHKRTLGL*	A*02:01	*89.8*	*1*	*Yes*/*yes*			
*99–108*	*GLSAMSTTDL*	A*02	*80.7*	*2*	*No*/*yes*			
*102–110*	*AMSTTDLEA*	A*02:01	*80.7*	*1*	*No*/*yes*			
15–23	VLCLRPVGA	A*02:01	88.4	1	No/no			
**8–16**	**QLDPARDVL**	A*02:01	**80.2**	**4**	**No/no**	**25 (1/4)**	**0 (0/20)**	**0 (0/10)**
115–123	CLFKDWEEL	A*02:01	39.9	1	Yes/yes			50 (3/6)
84–92	NAHQVLPKV	A*02:01	39.9	1	Yes/no			
97–105	TLGLAAMST	A*02:01	3.1	1	Yes/yes			
140–148	KLVCSPAPC	A*02:01	63.4	1	No/yes			50 (3/6)
44–53	VVPTDHGAHL	A*02:01	11.3	1	No/no			

a Epitopes are classified into different categories (gray/white areas) by ranking first on conservation [Cons (%)] and subsequently on functionality (Presence of functional amino acid/domain). Epitopes in italic type are preferred immunogenic targets because of their high conservation across HBV genotypes (conservation of >80%) and previous association with functional importance (Presence of functional amino acid/domain). The epitope in boldface type evokes a response in at least 25% of patients within a patient group, which was not significantly more prevalent than in other patient groups.

**TABLE 2:** Ranking of reported epitopes for polymerase based on conservation and function^*a*^

Amino acid positions	Amino acid sequence	HLA type(s)	Cons (%)	No. of papers reporting epitope	Presence of functional amino acid/domain	% responders (no. of responders/total no. of subjects tested)		
						Acute	Chronic	HCC
*389–397*	*VVDFSQFSR*	A*33, A*11, A*68:*01*	*99.8*	*3*	*Yes*/*yes*			
*388–397*	*LVVDFSQFSR*	A*11, A*33:*01*, A*68:*01*	*99.8*	*1*	*Yes*/*yes*	*5* (*1*/*22*)		
*746–755*	*GTDNSVVLSR*	A*11	*99.4*	*1*	*Yes*/*no*			
*374–383*	*FLVDKNPHNT*	A*02:*03*	*99.3*	*1*	*Yes*/*yes*			
*55–63*	*KVGNFTGLY*	A*03, A*11	*99.2*	*1*	*Yes*/*yes*	*9* (*2*/*22*)		
*368–378*	*RVTGGVFLVDK*	A*11	*99.1*	*1*	*Yes*/*yes*			
*369–378*	*VTGGVFLVDK*	A*03, A*11	*99.1*	*1*	*Yes*/*yes*			
*166–173*	*ASFCGSPY*	A*01:*01*, A*29:*02*, A*30:*02*	*97.3*	*1*	*Yes*/*yes*			
*166–175*	*ASFCGSPYSW*	B*58:*01*	*97.3*	*1*	*Yes*/*yes*			
***756–764***	***KYTSFPWLL***	A*24:*02*	***96.8***	***2***	***Yes*/*no***	*93* (*13*/*14*)*	***48* (*10*/*21*)**	
*147–156*	*YLHTLWKAGI*	A*02	*96.7*	*2*	*Yes*/*yes*			*0* (*0*/*10*)
*149–159*	*HTLWKAGILYK*	A*03, A*11, A*31:*01*, A*33:*01*, A*68:*01*	*96.7*	*1*	*Yes*/*yes*	*5* (*1*/*22*)		
*150–158*	*TLWKAGILY*	A*03, A*11	*96.7*	*1*	*Yes*/*yes*			
*150–159*	*TLWKAGILYK*	A*03, A*11	*96.7*	*1*	*Yes*/*yes*	*18* (*5*/*28*)		
*653–661*	*ALMPLYACI*	A*02:*01*, A*02:*02*, A*02:*03*, A*02:*04*, A*02:*06*	*96.4*	*4*	*Yes*/*no*	*20* (*4*/*20*)	*0* (*0*/*9*)	*0* (*0*/*10*)
*651–659*	*YPALMPLYA*	B*07:*02*, B*35:*01*, B*51, B*54:*01*	*96.4*	*1*	*Yes*/*no*	*0* (*0*/*12*)		
*365–374*	*TPARVTGGVF*	B*35, B*51	*95.5*	*1*	*Yes*/*yes*	*17* (*2*/*12*)		
*549–557*	*YMDDVVLGA*	A*02:*01*	*92.5*	*4*	*Yes*/*no*	*15* (*2*/*13*)	*0* (*0*/*21*)	*0* (*0*/*10*)
*549–558*	*YMDDVVLGAK*	A*03	*92.5*	*1*	*Yes*/*no*			
*789–797*	*DPSRGRLGL*	B*07:*02*	*91.0*	*1*	*Yes*/*no*			
*500–508*	*KLHLYSHPI*	A*02:*03*	*99.5*	*3*	*No*/*yes*			*0* (*0*/*10*)
*440–448*	*HPAAMPHLL*	B*07:*02*	*99.5*	*1*	*No*/*yes*	*0* (*0*/*12*)		
*47–55*	*NVSIPWTHK*	A*03, A*11, A*68:*01*	*98.3*	*1*	*No*/*yes*	*5* (*1*/*21*)		
*418–426*	*LLSSNLSWL*	A*02:*01*	*95.4*	*1*	*No*/*yes*			
*422–430*	*NLSWLSLDV*	A*02	*95.4*	*1*	*No*/*yes*			*0* (*0*/*10*)
*502–510*	*HLYSHPIIL*	A*02	*89.2*	*2*	*No*/*yes*			
770–778	WILRGTSFV	A*02	99.6	2	No/no			
**771–780**	**ILRGTSFVYV**	A*02:01	**99.6**	**2**	**No/no**	**43 (3/7)**	**0 (0/9)**	
531–539	SAICSVVRR	A*11, A*33:01, A*68:01	98.8	1	No/no	9 (2/22)		
**573–581**	**FLLSLGIHL**	A*02:01, A*02:06	**96.2**	**18**	**No/no**	59 (50/85)*	**13 (9/70)**	**0 (0/10)**
665–674	QAFTFSPTYK	A*03, A*11, A*68:01	95.9	2	No/no	14 (3/21)		
524–533	FLLAQFTSAI	A*02:01	94.4	2	No/no			0 (0/10)
525–533	LLAQFTSAI	A*02	94.4	1	No/no			0 (0/10)
541–550	FPHCLAFSYM	B*07:02, B*35:01, B*51, B*53:01, B*54:01	92.5	1	No/no	0 (0/12)		
623–631	PVNRPIDWK	A*03, A*11	91.1	1	No/no			
763–771	LLGCAANWI	A*02:01	85.0	1	No/no			
61–69	GLYSSTVPV	A*02:01	57.7	1	Yes/yes	8 (1/12)		
485–493	NLYVSLLLL	A*02:01	52.3	1	Yes/yes			
744–752	IIGTDNSVV	A*02:01	17.1	1	Yes/no			
361–369	RIPRTPSRV	A*02	4.1	1	Yes/yes			
338–346	CLSLIVNLL	A*02	3.9	1	Yes/yes			
651–659	YPALMPLSA	B*54:01	0.0	1	Yes/no			
**453–461**	**GLSRYVARL**	A*02:01, A*02:02, A*02:03	**68.3**	**12**	**No/yes**	50 (39/78)*	**6 (5/87)**	**0 (0/10)**
453–461	GLPRYVARL	A*02:01, A*02:07	30.8	1	No/yes			
466–474	RIINNQHRT	A*02:01	24.3	1	No/yes			
261–269	GSGPTHNCA	A*11:01	43.7	1	No/no			
**814–822**	**SLYADSPSV**	A*02:01	**43.3**	**5**	**No/no**	25 (11/44)*	**0 (0/22)**	
671–679	PTYKAFLSK	A*11:01	31.4	1	No/no			
796–804	GLSRPLLRL	A*02	21.3	1	No/no			
573–581	FLLSLGIHI	A*02	0.2	1	No/no			

a Epitopes are classified into different categories (gray/white areas) by ranking first on conservation [Cons (%)] and subsequently on functionality (Presence of functional amino acid/domain). Epitopes in italic type are preferred immunogenic targets because of their high conservation across HBV genotypes (conservation of >80%) and previous association with functional importance (Presence of functional amino acid/domain). Epitopes in boldface type evoke a response in at least 25% of patients within a patient group, which was significantly more prevalent than in the other patient groups in 4 cases (asterisks).

We argued that epitopes in which the least conserved amino acid was still present in 80% of all sequences tested would be targetable in the majority of the population. Combining this criterion with the preference for functional association, we found 7 HBx-derived and 26 Pol-derived sequences reported as epitopes across HLA types to be preferential targets for global immunotherapy (italic type in Tables 1 and 2, respectively). If documented, Tables 1 and 2 additionally show response percentages and frequencies in acute/resolved HBV infection and HBV-related HCC for all HBx- and Pol-derived epitopes reported in Hepitopes. Patients suffering from acute infection can clear the disease spontaneously, implying that HBV-specific T cells frequently identified in acute patients or resolved individuals may have contributed to viral clearance. Thus, such responses may be of particular interest to boost or induce in chronic patients. For peptides extracted from Hepitopes that evoke a response in at least 25% of patients (Tables 1 and 2, boldface type), we identified 4 Pol-derived sequences against which responses were significantly more prevalent in patients with acute or resolved infection (Table 2, asterisks).

### Prediction of novel HLA-I-binding peptides derived from HBx and polymerase.

To extend epitope coverage within the infected population, we set out to identify novel peptides that can bind at least 1 out of 6 HLA supertypes prevalent in Caucasian, African, or Asian populations for which *in vitro* assays to confirm binding were also at our disposal (i.e., supertypes HLA-A*01, -A*02, -A*03, -A*24, -B*07, and -B*08). We first predicted binders spanning 8 to 14 amino acids for supertype-representative HLA types using the established *in silico* prediction tool NetMHCpan to make a frequency distribution of predicted binders (Fig. 2 and 3, gray bar diagrams). The densities of all predicted binders per amino acid were similar between Pol and HBx (means ± standard deviations [SD] of 16.36 ± 12.62 for Pol and 15.60 ± 9.49 for HBx; *P* = 0.57 by a Mann-Whitney test). Predicted binders spanning 9 to 11 amino acids were subsequently aligned to our maps outlining conservation and function (Fig. 2 and Fig. S2) since 9- to 11-mers are most likely to represent an epitope (70). Individual peptide positions for Pol are exclusively shown in Fig. S2 to enable high-resolution zoom-in on peptides of interest, which would not be possible in Fig. 3.

**FIG 4:**
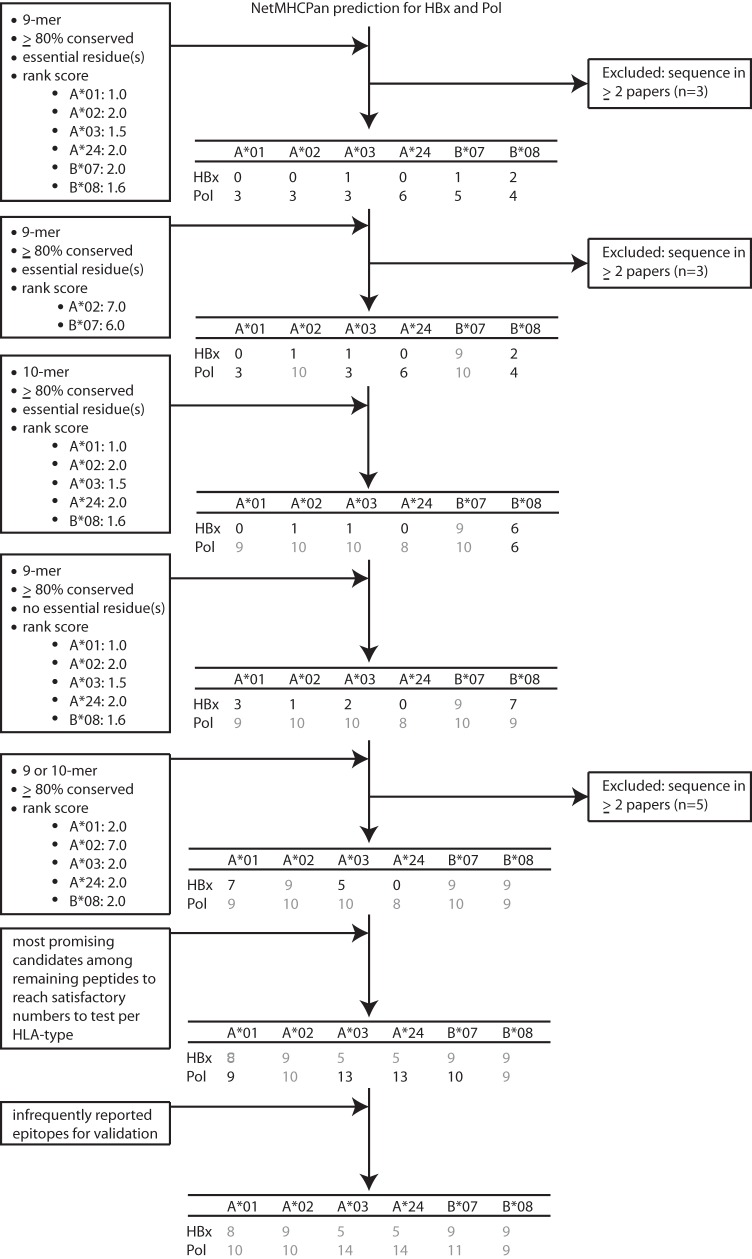
Selection of peptide candidates to test in an *in vitro* HLA binding assay. Selection was based on conservation, functionality, peptide length, and rank score. Either the default rank score of ≤2.0 or an adjusted value for the appropriate HLA type was used based on past performance of the *in vitro* binding assay (see Fig. S3 in the supplemental material), as indicated in the sequential diagram steps. Peptides that were predicted to bind several HLA supertype representatives were prioritized throughout the selection procedure in case the number of peptide candidates exceeded 8 for each HLA type. After each step, we excluded epitopes that we considered established (reported in a minimum of 2 papers) using the Hepitopes database. Gray numbers in the central peptide table represent the conditions for which inclusion was not further pursued.

**FIG 5:**
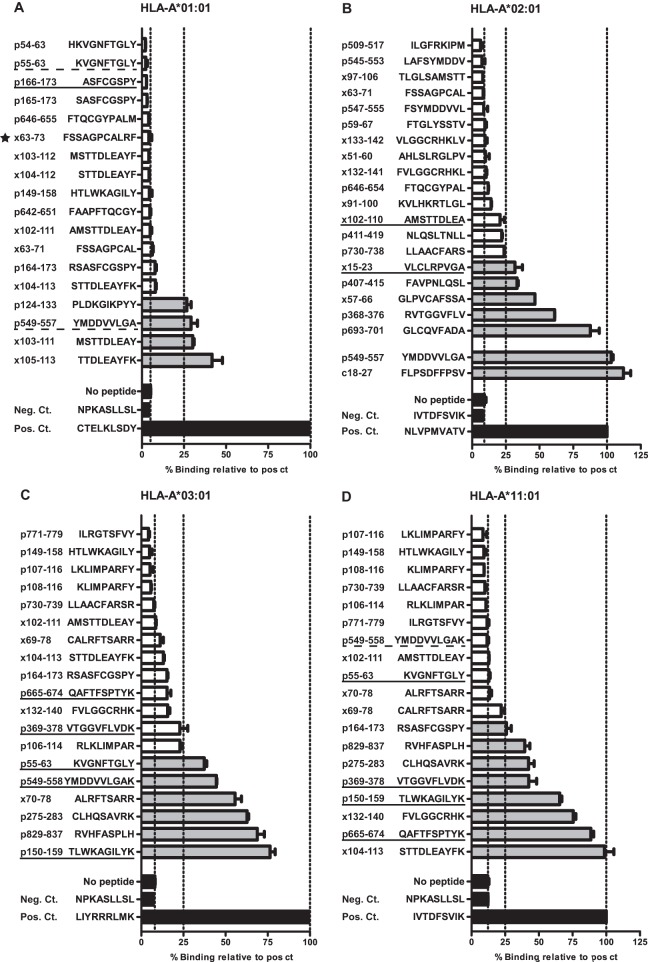

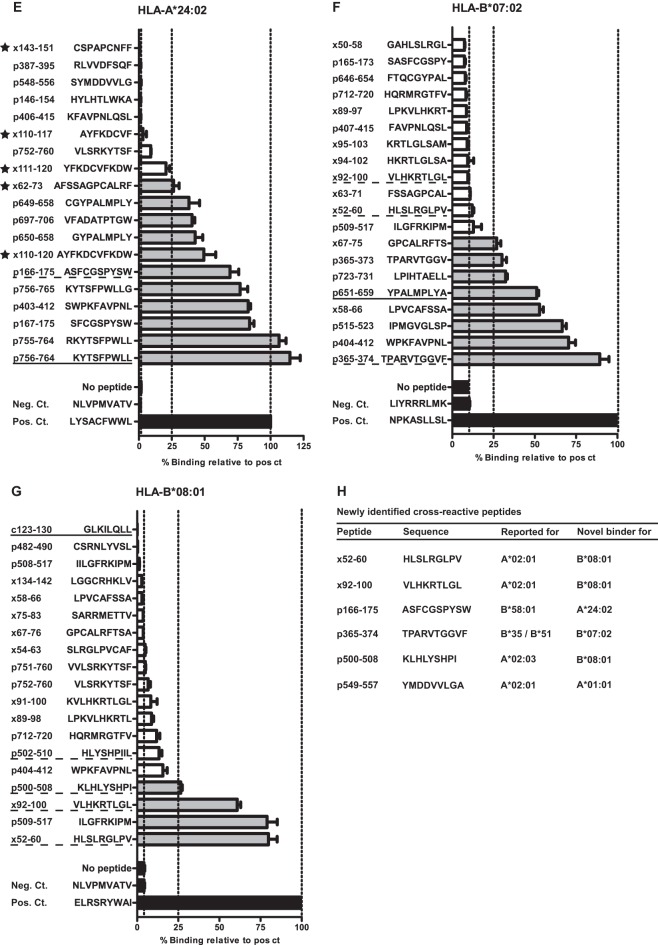
Binding capacities of all preselected peptide candidates. Binding of predicted peptide candidates is represented as percent binding of positive-control peptides (Pos. Ct.), which have a high affinity for the indicated HLA type. Means and standard deviations are depicted for controls (black), binders (>25% of the positive control) (gray), and nonbinders (≤25% of the positive control) (white). As a negative control (Neg. Ct.), we included a known nonbinding peptide for each HLA type and a condition under which no peptide was present. Solid underlined peptides are infrequently described as epitopes for the HLA type tested. Dotted underlined peptides have so far been described only as an epitope for another HLA type, of which cross-reactive binders are summarized in the table. Stars indicate the peptides that did not meet our length and conservation thresholds.

The prediction yielded totals of 251 potential novel HLA binders for HBx and 1,655 for Pol (Fig. 1, right), including both weak and strong binders (i.e., predicted NetMHCpan rank scores based on past performance [Fig. S3]). Of these, we selected the most promising peptides for validation of HLA binding in an *in vitro* UV-based assay (71). For practical and economic reasons, we aimed to test the binding of 96 unique peptide sequences over both proteins and across HLA types. We included 2 well-described HLA-A*02 epitopes from HBcAg and Pol (c18-27 and p549-557, respectively) to put the binding capacity of our newly identified binders into context. For other HLA types, we also aimed to include a known HBV epitope for comparison, but only less established epitopes reported once or twice were available (see Fig. 5, solid underlining). This also included c123-130 since this was the only HBV-derived epitope registered in Hepitopes for HLA-B*08. Potential binders were prioritized based on peptide length (9-mers preferred), predicted HLA-binding strength, conservation, and functional importance of included amino acids (Fig. 4). For HLA-A*01 and HLA-A*24, there was an unsatisfactory number of predicted binders for HBx to maintain our strict thresholds for conservation and peptide length. For these conditions, we therefore also included some less conserved peptides or peptides spanning 8 to 12 amino acids (Fig. 5, stars, and Table S3). Furthermore, we selected several additional peptides that were infrequently (once or twice) reported as an epitope in the literature and therefore considered unestablished (Fig. 5, solid underlining). Moreover, peptides that were predicted to bind several HLA types were prioritized throughout the selection procedure, which led to a total set of 113 potential peptide-HLA combinations to test in an *in vitro* binding assay: 45 for HBx and 68 for Pol. The majority of the peptides mapped to highly conserved areas with established functional importance. The median conservation scores among selected peptides were above 93% for HBx and even above 96% for Pol (Fig. S4).

### *In vitro* binding capacity of preselected peptides derived from HBx and polymerase.

Next, the HLA-binding capacity of selected peptides was tested in a UV-based *in vitro* binding assay. Peptides were classified as HLA binders when their binding capacity was higher than 25% of that of a known high-affinity peptide (Table S4). HLA-A*11:01 and HLA-A*03:01 were both tested as members of the HLA-A*03 supertype since many HBV-infected patients are Asian and HLA-A*11:01 is more prevalent in this population than the supertype representative HLA-A*03:01, which is more prevalent in Caucasians (72). We identified 13 binders for HBx and 33 for Pol across HLA supertypes, including novel binders that have been described previously in the context of another HLA type (Fig. 5A to G, dotted underlining, and Fig. 5H). Notably, both HBx- and Pol-derived binders were identified for each HLA supertype tested. For HLA-A*02, the well-established epitopes c18-27 and p549-557 scored even better than the positive control (Fig. 5B). In contrast, binding of infrequently reported epitopes (Fig. 5, solid underlining) could not always be verified.

### Immunogenicity of selected HLA-binding peptides.

**FIG 6:**
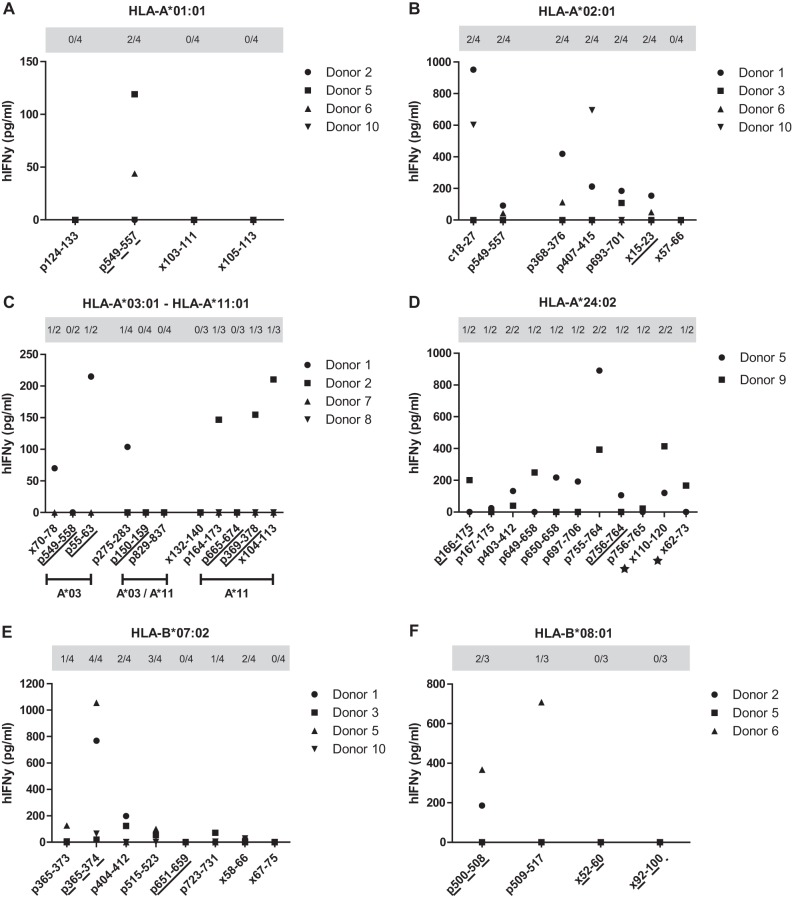
Immunogenicity of HLA-binding peptides. DMSO-subtracted IFN-γ concentrations produced by expanded PBMCs from 9 HBV resolvers were measured in response to all HLA binders identified, including the well-established c18-27 and p549-557 epitopes (B). Gray boxes present the number of responsive donors as a fraction of the total number of subjects tested for each peptide. Solid underlined HLA binders are infrequently described as epitopes for the HLA supertype tested. Dotted underlined HLA binders have so far been described only as an epitope for another HLA supertype. Stars indicate the peptides that did not meet our length and/or conservation thresholds.

Finally, the immunogenicity of all HBx- and Pol-derived binders was assessed to determine which binders would be most interesting for the development of an antigen-based HBV-targeting immunotherapy. Peripheral blood mononuclear cells (PBMCs) from blood donors who had previously resolved an HBV infection were expanded in the presence of peptide pools, followed by single-peptide restimulation and an interferon gamma (IFN-γ) enzyme-linked immunosorbent assay (ELISA). As expected, IFN-γ production was detected in response to the well-established epitopes c18-27 and p549-557 (Fig. 6B). Furthermore, IFN-γ production was highly variable, and some donors generally seemed to respond better than others (Fig. 6). In total, we observed responses against 5 completely novel HBx- and 17 novel Pol-derived peptides. Additionally, we observed IFN-γ production in response to 1 HBx- and 3 Pol-derived less established epitopes, although none of these responses were very high (Fig. 6, solid underlining). Importantly, 4 additional Pol-derived peptides elicited responses in donors negative for the HLA type in which these peptides previously yielded epitopes (Fig. 6, dotted underlining, and Table 3). Finally, there was no measurable response to 6 Pol-derived and 7 HBx-derived HLA binders in any of the donors tested (Fig. 6, gray boxes). Table 4 shows our main findings, in which all prioritized HBx- and Pol-derived epitopes from Tables 1 and 2 are categorized according to the HLA (super)types of interest with reference to all HLA types for which they were described. Only epitope p166-175 was prioritized in our analysis but was not described for any of the HLA (super)types of interest. Table 4 also includes all peptides against which IFN-γ responses were detected.

**TABLE 3:** HLA-I types of HBV resolver donors used for immunogenicity testing of HLA binders

Donor	HLA-A type	HLA-B type	HLA-C type
1	03:01	07:02	07:02
	02:01	07:02	07:02

2^*a*^	01	08	06
	11	13	07

3	02:01	18:01	12:03
	25:01	07:02	07:02

5	01:01	08:01	07:01
	24:02	07:02	07:02

6	01:01	08:01	07:01
	02:01	40:01	03:04

7	03:01	52:01	02:02
	11:01	51:01	12:02

8	23:01	49:01	07:01
	11:01	18:01	12:02

9^*a*^	24	15	01
	31	22	14

10	01:01	07:02	07:01
	02:01	44:02	07:02

a The HLA type is available in 2-digit resolution only.

**TABLE 4:** Recommendation of the most potent T cell epitopes classified by HLA (super)type

HLA type^*b*^	Reported epitope			Novel/validated epitope^*a*^		
	Designation	Sequence	HLA (super)type(s)	Designation	Sequence	HLA (super)type
A*01	p166-173	ASFCGSPY	A*01:01, A*29:02, A*30:02	*p549-557*	*YMDDVVLGA*	A*01:01

A*02	p147-156	YLHTLWKAGI	A*02	p368-376	RVTGGVFLV	A*02:01
	p374-383	FLVDKNPHNT	A*02:03	p407-415	FAVPNLQSL	A*02:01
	p418-426	LLSSNLSWL	A*02:01	p693-701	GLCQVFADA	A*02:01
	p422-430	NLSWLSLDV	A*02	x15-23	VLCLRPVGA	A*02:01
	p500-508	KLHLYSHPI	A*02:03			
	p502-510	HLYSHPIIL	A*02			
	p549-557	YMDDVVLGA	A*02:01			
	p653-661	ALMPLYACI	A*02:01, A*02:02, A*02:03, A*02:04, A*02:06			
	x52-60	HLSLRGLPV	A*02:01			
	x91-100	KVLHKRTLGL	A*02:01			
	x92-100	VLHKRTLGL	A*02:01			
	x99-108	GLSAMSTTDL	A*02			
	x102-110	AMSTTDLEA	A*02:01			
	x133-141	VLGGCRHKL	A*02:01			

A*03	p47-55	NVSIPWTHK	A*03, A*11, A*68:01	p275-283	CLHQSAVRK	A*03:01
	p55-63	KVGNFTGLY	A*03, A*11	p55-63	KVGNFTGLY	A*03:01
	p149-159	HTLWKAGILYK	A*03, A*11, A*31:01, A*33:01, A*68:01	x70-78	ALRFTSARR	A*03:01
	p150-158	TLWKAGILY	A*03, A*11			
	p150-159	TLWKAGILYK	A*03, A*11			
	p369-378	VTGGVFLVDK	A*03, A*11			
	p388-397	LVVDFSQFSR	A*11, A*33:01, A*68:01			
	p389-397	VVDFSQFSR	A*33, A*11, A*68:01			
	p549-558	YMDDVVLGAK	A*03			

A*11	p47-55	NVSIPWTHK	A*03, A*11, A*68:01	p164-173	RSASFCGSPY	A*11:01
	p55-63	KVGNFTGLY	A*03, A*11	p369-378	VTGGVFLVDK	A*11:01
	p149-159	HTLWKAGILYK	A*03, A*11, A*31:01, A*33:01, A*68:01	x104-113	STTDLEAYFK	A*11:01
	p150-158	TLWKAGILY	A*03, A*11			
	p150-159	TLWKAGILYK	A*03, A*11			
	p368-378	RVTGGVFLVDK	A*11			
	p369-378	VTGGVFLVDK	A*03, A*11			
	p388-397	LVVDFSQFSR	A*11, A*33:01, A*68:01			
	p389-397	VVDFSQFSR	A*33, A*11, A*68:01			
	p746-755	GTDNSVVLSR	A*11			
	x135-143	GGCRHKLVC	A*11:01			

A*24	p756-764	KYTSFPWLL	A*24:02	*p166-175*	*ASFCGSPYSW*	A*24:02
				p167-175	SFCGSPYSW	A*24:02
				p403-412	SWPKFAVPNL	A*24:02
				p649-658	CGYPALMPLY	A*24:02
				p650-658	GYPALMPLY	A*24:02
				p697-706	VFADATPTGW	A*24:02
				p755-764	RKYTSFPWLL	A*24:02
				p756-764	KYTSFPWLL	A*24:02
				p756-765	KYTSFPWLLG	A*24:02
				x62-73	AFSSAGPCALRF	A*24:02
				x110-120	AYFKDCVFKDW	A*24:02

B*07	p365-374	TPARVTGGVF	B*35, B*51	p365-373	TPARVTGGV	B*07:02
	p440-448	HPAAMPHLL	B*07:02	*p365-374*	*TPARVTGGVF*	B*07:02
	p651-659	YPALMPLYA	B*07:02, B*35:01, B*51, B*54:01	p404-412	WPKFAVPNL	B*07:02
	p789-797	DPSRGRLGL	B*07:02	p515-523	IPMGVGLSP	B*07:02
				p723-731	LPIHTAELL	B*07:02
				x58-66	LPVCAFSSA	B*07:02

B*08				*p500-508*	*KLHLYSHPI*	B*08:01
				p509-517	ILGFRKIPM	B*08:01

B*58	p166-175	ASFCGSPYSW	B*58:01			

a Validated for the tested HLA type (underlining)/tested for an HLA type other than the one previously reported (italics).

b Boldface HLA type indicates groups of epitopes with preference for this particular HLA type in the corresponding gray/white area.

## DISCUSSION

The aim of this study was to rationally address two major hurdles in developing a generic antigen-based immunotherapy for HBV: (i) the lack of prioritization of epitopes in further studies toward clinical implementation and (ii) the shortage of non-HLA*02-restricted epitopes.

To address the first issue, we ranked all currently described HBx- and Pol-derived epitopes according to conservation and association with viral indispensability. Conservation patterns were similar to those previously reported, with the most conspicuous observation being that the spacer domain of Pol is extremely variable (27, 42, 73). We obtained all reported HBx- and Pol-derived epitopes used for this study from the Hepitopes database, which also contains less firmly established epitopes. Thus, our ranking might contain epitopes that need further validation prior to implementation in immunotherapies.

Four Pol-derived epitopes were more frequently described in acute or resolved infection than in chronic infection. Of these, p756-764 and p573-581 scored the highest in conservation. p756-764 contains at least 1 amino acid important for viral persistence, but no functional relevance has been described for p573-581. Since the DNA sequence coding for p573-581 completely overlaps that of HBsAg, we interrogated the literature for the functional relevance of amino acids in this overlapping part of HBsAg. However, no functional association was reported (73–75). Although T cell responses to HBsAg are frequently deleted/exhausted and potentially difficult to revive because of antigen overstimulation (76–78), this does not necessarily translate to Pol since HBsAg and Pol are not produced at equal levels. Besides, HBsAg and Pol are derived from different open reading frames (ORFs), which results in a different amino acid sequence for HBsAg than for Pol despite being based on largely the same DNA sequence (79). Thus, although not yet linked to function, the high conservation score still nominates p573-581 for utilization in generic immunotherapy. In contrast, the other two epitopes that were highly prevalent in acute patients, p453-461 and p814-822, were much less conserved (68.3% and 43.3%, respectively) and did not contain any amino acids with demonstrated functional relevance. These epitopes might therefore be less interesting for the development of global immunotherapy.

Although we rationalized to prioritize conserved peptide sequences, we reckon that regions containing prevalent sequence variation could still be interesting if immunogenicity is preserved. Novel T cell responses may arise due to cross-reaction between the variant and the original sequence (80, 81). This could, e.g., be true for p453-461, of which 2 sequence variants are described as epitopes in the same HLA context. However, whether a variation can induce cross-reactive T cells needs to be experimentally assessed for each peptide sequence and its variant individually, which is beyond the scope of our study.

The second issue in developing generic antigen-specific immunotherapy for HBV is that non-HLA-A*02-restricted epitopes are vital but scarce. Here, we identified novel epitopes for 6 HLA supertypes most prevalent in the HBV-infected population. Supertypes HLA-A*02, HLA-A*03, and HLA-B*07 altogether cover >85% of the Caucasian, African, and Asian populations (23). HLA-A*24 further extends coverage to the Asian population, whereas HLA-A*01 and HLA-B*08 extend to the Caucasian population. For all these HLA supertypes combined, the predicted binder quantity was clearly higher for Pol than for HBx. This was mostly because HBx is smaller (154 amino acids) than Pol (843 amino acids) since we found similar densities of predicted binders for both proteins. Opposed to this observation, Pol, out of all HBV-derived proteins, previously showed the highest density of predicted CD8^+^ epitopes, while HBx seemed more subject to immune-pressure-induced deletion of epitopes (82). However, that study included only 107 HBV-derived sequences of a single genotype, whereas our study includes more than 7,000 sequences for each protein with all genotypes represented.

In the present study, prediction was performed using an established, well-performing *in silico* tool. However, it cannot be excluded that potent epitopes remain unidentified by using this approach. Due to limited resources, we could include only the most potent peptide candidates, leaving many unexplored. In addition, we have not further studied functionality in overlapping ORFs with respect to Pol and HBx that may yield even more epitopes with a low chance of immune escape. However, unexplored epitopes may still be identified, e.g., by using mass spectrometry on HLA-eluted peptides (83). We additionally demonstrate that prediction yields many false-positive HLA binders, highlighting the necessity of validation assays. To investigate which HLA binders would be relevant for future studies, immunogenicity was assessed in subjects who resolved HBV infection. We performed antigen-specific expansion to allow detection of responses that might be low if HBV was cleared a long time ago. Previous reports showed that functional HBcAg-specific CD8^+^ T cells were significantly less abundant in patients who cleared HBV infection long ago than in patients who cleared infection more recently (84, 85). Indeed, some donors generally gave a stronger IFN-γ response than others, which might reflect more recent clearance. Thus, high-level IFN-γ production in our experiments might not directly translate to strong immunogenicity. Inversely, epitopes yielding low IFN-γ responses should not be immediately disregarded as promising.

Recent reports showed that Pol cognate T cells might be more exhausted in terms of phenotype and function than HBcAg-directed T cells (39, 40, 86). Nonetheless, as also noted by Bertoletti and Kennedy (87), this does not dismiss Pol as a suitable target for immunotherapy. First of all, Pol-derived epitopes have a role in viral control after discontinuation of antiviral therapy (36). Second, data were based on HBeAg-negative patients in whom HBeAg-mediated exhaustion of HBcAg T cells due to overlapping sequences between HBeAg and HBcAg may have been (partially) reverted (88). The fact that HBcAg immunity may be of relatively good quality in HBeAg-negative patients, however, renders HBcAg an interesting target for immunotherapy, especially in this patient group. Finally, the above-mentioned papers primarily focused on only 1 or 2 epitopes. Thus, the conclusion that Pol-directed T cells in general are more exhausted than HBcAg cognate T cells should be taken with prudence and needs further investigation for more epitopes across different HLA types. The epitopes put forward in our study would make good tools for such efforts.

We identified a few novel epitopes for which the sequence overlaps that of an epitope that has previously been described for the HLA type tested. This is most apparent for the novel epitopes p755-764 and p365-373, of which p755-764 resulted in superior IFN-γ production compared to the known epitope p756-764 in both donors tested. It would now be interesting to investigate how responses to these epitope variants relate in CHB patients, especially since p755-764 has been the only HBV-derived epitope described for supertype HLA-A*24 until very recently. For HLA-A*24:02, we studied the binding capacity of 3 peptides that were recently assessed for the first time in another study (89). Those authors did not detect cytotoxic T cells against p146-154 and p387-395, which fits our finding that neither peptide bound HLA-A*24:02. Conversely, we observed a response to p650-658 in 1 of the 2 HLA-A*24:02-positive donors, whereas none of the 3 HBV resolvers described by Yamamiya et al. responded. This difference may be due to the low number of resolvers tested and emphasizes the need to validate the novel epitopes described here in more subjects prior to implementation in immunotherapies. In addition, further characterization of cognate T cell populations in different patient populations *ex vivo* is desired to determine which epitope has true clinical potential. Because we identified epitopes for 6 HLA supertypes that have limited overlap, this would require vast numbers of difficult-to-obtain samples, which is beyond the scope of this paper. Our immunogenicity assays aimed to explore which novel HLA binders had the intrinsic potential to boost immune responses, and we have delivered 30 epitopes that now provide a rational starting point for more elaborate efforts.

Although this study focused on CD8^+^ T cell epitopes, we recognize the importance of CD4^+^ T cell and B cell responses in viral clearance. Importantly, for studies pursuing HLA-II targets, the maps generated here detailing where HBx and Pol are most conserved and vulnerable to immune attack are also highly relevant. As such, they might aid in the design of synthetic long peptide (SLP) vaccines. SLPs can be designed to harbor both HLA-I and HLA-II epitopes and are processed more efficiently by dendritic cells than whole proteins (90). Importantly, SLPs directed against human papillomavirus (HPV)-induced neoplasms and malignancies have already been proven successful in clinical trials (91), and we have previously reported that SLPs show promise for use in HBV patients (92). The present study especially facilitates SLP design through the identification of immunogenic sequences that were previously described as epitopes in the context of a different HLA type than the ones tested here. For example, p365-374 was previously reported for HLA-B*35 and HLA-B*51 but now gave a response in 4/4 donors positive for HLA-B*07. The phenomenon that one peptide sequence can yield an epitope in several HLA supertypes has been described for other viral sequences (93) and opens up the interesting possibility of targeting a broad proportion of the infected population with just a single amino acid sequence. Because of this high population coverage, sequences yielding epitopes in multiple HLA types may be particularly interesting targets to include in different forms of immunotherapy such as SLP vaccination. In addition, our comprehensive analysis of HBx and Pol also aids in the development of T cell therapies by allowing selection of T cell receptors (TCRs).

In conclusion, we provide a rational methodology for the selection and discovery of the most potent HBV-derived HLA-I T cell epitopes. In addition, we propose novel T cell epitopes for a broad range of HLA (super)types covering the vast majority of the HBV-infected population that target the virus where it is most vulnerable. Collectively, the results of this study provide a valuable resource to guide future development of HBV-specific immunotherapies.

## MATERIALS AND METHODS

### Peptide prediction and selection.

A frequency table was downloaded from HBVdb V42.0 (50, 51) based on HBV sequences of all genotypes for HBx (*n* = 8,127) and Pol (*n* = 7,489). Positions where a gap (indicated by “−”) was most frequent were deleted, after which the dominating amino acid at each position was determined. Percentages of sequences containing the dominant amino acid were calculated as the conservation score. Combining all dominant amino acids for Pol led to the consensus sequence MPLSYQHFRKLLLLDDEAGPLEEELPRLADEGLNRRVAEDLNLGNLNVSIPWTHKVGNFTGLYSSTVPVFNPEWQTPSFPDIHLQEDIINRCQQFVGPLTVNEKRRLKLIMPARFYPNVTKYLPLDKGIKPYYPEHVVNHYFQTRHYLHTLWKAGILYKRETTRSASFCGSPYSWEQELQHGRLVFQTSKRHGDESFCSQSSGILSRSPVGPCIQSQLKQSRLGLQPQQGSLARRQQGRSGSIRARVHPTTRRSFGVEPSGSGHIDNSASSSSSCLHQSAVRKAAYSHLSTSKRQSSSGHAVELHNIPPSSARSQSEGPVFSCWWLQFRNSKPCSDYCLSHIVNLLEDWGPCTEHGEHHIRIPRTPARVTGGVFLVDKNPHNTTESRLVVDFSQFSRGNTRVSWPKFAVPNLQSLTNLLSSNLSWLSLDVSAAFYHLPLHPAAMPHLLVGSSGLSRYVARLSSNSRIINNQHGTMQNLHDSCSRNLYVSLLLLYKTFGRKLHLYSHPIILGFRKIPMGVGLSPFLLAQFTSAICSVVRRAFPHCLAFSYMDDVVLGAKSVQHLESLYTAVTNFLLSLGIHLNPNKTKRWGYSLNFMGYVIGSWGTLPQEHIVQKIKQCFRKLPVNRPIDWKVCQRIVGLLGFAAPFTQCGYPALMPLYACIQAKQAFTFSPTYKAFLCKQYLNLYPVARQRPGLCQVFADATPTGWGLAIGHQRMRGTFVAPLPIHTAELLAACFARSRSGAKLIGTDNSVVLSRKYTSFPWLLGCAANWILRGTSFVYVPSALNPADDPSRGRLGLYRPLLRLPFRPTTGRTSLYAVSPSVPSHLPDRVHFASPLHVAWRPP.

The resulting consensus sequence for HBx was determined to be MAARLCCQLDPARDVLCLRPVGAESRGRPLSGPLGTLPSPSPSAVPADHGAHLSLRGLPVCAFSSAGPCALRFTSARRMETTVNAHQVLPKVLHKRTLGLSAMSTTDLEAYFKDCVFKDWEELGEEIRLKVFVLGGCRHKLVCSPAPCNFFTSA.

These sequences were loaded into NetMHCpan3.0 (94) to predict binders for HLA supertype representatives HLA-A*01:01, HLA-A*02:01, HLA-A*03:01, HLA-A*24:02, HLA-B*07:02, and HLA-B*08:01. Furthermore, the cumulative frequency of each amino acid in any predicted HLA binder was calculated. Predicted HLA binder densities were compared between Pol and HBx using a two-tailed Mann-Whitney test. Amino acid sequences of reported functional domains were aligned with the consensus sequence using the NCBI tool COBALT (95). Similarly, amino acids that alone or in combination were previously associated with a loss of viral replication were aligned to the consensus sequence. Functionally associated amino acids were more numerous for Pol than for HBx. Therefore, an additional threshold of a ≥50% loss of viral persistence for Pol was introduced to select the most crucial amino acids. Tables S1 and S2 in the supplemental material present literature references on functional domains and amino acids. The most promising predicted binders were selected for each protein and HLA type separately. Binding of the selected peptides was subsequently validated in an *in vitro* binding assay as described below.

### *In vitro* HLA binding validation.

Synthetic peptides (Peptide 2.0 Inc.) of selected potential HLA binders were used in an *in vitro* binding assay as described previously (96). In brief, peptide exchange reactions were performed by exposure for 30 min of conditional peptide-HLA complexes (pHLA) (0.53 μM) to long-wavelength UV using a 366-nm UV lamp (Camag) in the presence or absence of the indicated peptide (50 μM). Subsequently, the peptide exchange efficiency was analyzed using an HLA-I enzyme-linked immunosorbent assay (ELISA), which detects beta-2-microglobulin of peptide-stabilized HLA-I complexes in an exchange reaction mixture. To this end, streptavidin (2 μg/ml) was bound to polystyrene microtiter wells (Nunc MaxiSorp). After washing and blocking, the HLA complex present in exchange reaction mixtures or controls was captured by the streptavidin on the microtiter plate via its biotinylated heavy chain (incubation for 1 h at 37°C). Nonbound material was removed by washing. Subsequently, horseradish peroxidase (HRP)-conjugated antibody to human beta-2-microglobulin (0.6 μg/ml; Sanquin Reagents BV) was added (incubation for 1 h at 37°C). After the removal of the nonbound HRP conjugate by washing, an ABTS [2,2′-azino-bis(3-ethylbenzothiazoline-6-sulfonic acid)diammonium salt] (Sanquin Reagents BV) substrate solution was added to the wells. The reaction was stopped after 8 min (incubation at room temperature) by the addition of a 2% (wt/vol) oxalic acid dihydrate stop solution (Sanquin Reagents BV) and read in a Thermo Electron Multiskan Ascent ELISA reader at 414 nm. Every peptide was independently exchanged twice. Every exchange mixture was measured in duplicate by the HLA-I ELISA. The absorbances of all the peptides were normalized to the absorbance of a known HLA allele-specific ligand with high affinity for each corresponding allele (representing 100%) (Table S4). Negative controls included an HLA allele-specific nonbinder (Table S4) and UV irradiation of the conditional HLA-I complex in the absence of a rescue peptide.

### Determining immunogenicity.

Peptides with >25% binding in the *in vitro* HLA binding assay were assessed for immunogenicity. Briefly, PBMCs were isolated by Ficoll (GE Healthcare) density centrifugation from buffy coats of 9 donors who had previously resolved HBV infection. Buffy coats were provided by the local blood bank with corresponding 2-digit HLA types. Four-digit HLA typing was performed for 7 out of 9 donors using the global screening array (GSA) (Illumina through the Human Genomics Facility, Erasmus MC Rotterdam) (Table 3). All donors gave written informed consent. PBMCs were cultured in Iscove’s modified Dulbecco’s medium (IMDM) (Lonza) containing 2% human serum (Sanquin) and 50 IU/ml human interleukin-2 (hIL-2) (Miltenyi) in the presence of peptide pools of a maximum of 5 peptides of interest based on HLA matching at 10 μg/ml/peptide. After 14 days, 200,000 cells were restimulated with the peptides of interest for 48 h at 37°C with 10 μg/ml/peptide in triplicate. Supernatants from restimulations were subsequently used in a human IFN-γ (hIFN-γ) ELISA (BioLegend) according to the manufacturer’s instructions. Plates were read at a 450-nm wavelength using an Infinite 200Pro ELISA reader. hIFN-γ levels were calculated from background-subtracted optical density (OD) values (means from triplicates) using a supernatant derived from a previously successful restimulation with c18-27 that was quantified in a separate ELISA using the hIFN-γ standard provided by the manufacturer. HLA binders with a mean OD value of at least the mean plus 2 times the standard deviation (SD) of the dimethyl sulfoxide (DMSO) control were quantified. HLA binders that did not meet this criterion were included as nonresponsive (0 pg/ml IFN-γ produced). HLA binders against which IFN-γ production was detected in at least one donor tested were classified as epitopes.

### Comparison of response frequencies of described epitopes in acute versus chronic patients.

The Hepitopes initiative previously performed an extensive literature search of the Medline and Embase databases to collect all HBV-derived HLA-I epitopes from 112 papers (24, 49). All HBx- and Pol-derived epitopes reported in the Hepitopes database that were identified in human hosts were subjected to NCBI protein BLAST analysis to verify amino acid positions in the consensus sequence. Presented conservation scores apply to the least conserved amino acid within an epitope. Epitopes that harbor a minimum of one amino acid essential for viral replication or that completely span a functional domain are scored with “yes” for “amino acid” or “domain,” respectively. Epitopes are classified into different categories (gray/white areas) by ranking first on conservation and subsequently on functionality. Epitopes with equal scores for both parameters are ranked on the number of papers in which they were mentioned and subsequently on the number of HLA types for which they were identified. All corresponding papers and their supplemental material as listed in the Hepitopes database were checked to acquire the cumulative number of chronic, acute/resolved, and HBV-positive HCC patients responding to each epitope (42, 55–85). A responsive patient was defined as an individual with either positive multimer staining, IFN-γ production in an enzyme-linked immunosorbent spot (ELISPOT) assay, or a combination of these readouts. When peptide pools were used and no responses were found, all peptides tested in that pool were considered to have given a negative result and were included as such. When peptide pools were used and responses were found while it was unclear which epitope caused the response, all peptides tested in that pool were excluded from the analysis for that particular reference. For epitopes evoking a response in at least 25% of patients in a particular patient group, responses were compared between patient groups using a two-tailed Fisher exact test followed by the Holms multiple-testing correction (97) and found significant when the *P* value was <0.05. For the few epitopes with a response in at least 25% of HCC patients, patient groups could not be compared due to the lack of data for CHB and acute hepatitis patients.

## Supplementary Material

Supplemental file 1
