# Supplementary material for: Discovery and Selection of Hepatitis B Virus-Derived T Cell Epitopes for Global Immunotherapy Based on Viral Indispensability, Conservation, and HLA-Binding Strength
Source: J Virol. 2020 Mar 17;94(7):e01663-19. doi: 10.1128/JVI.01663-19 (PMC7081907; doi:10.1128/JVI.01663-19)
Supplement: Supplemental file 1 [file JVI.01663-19-s0001.pdf]

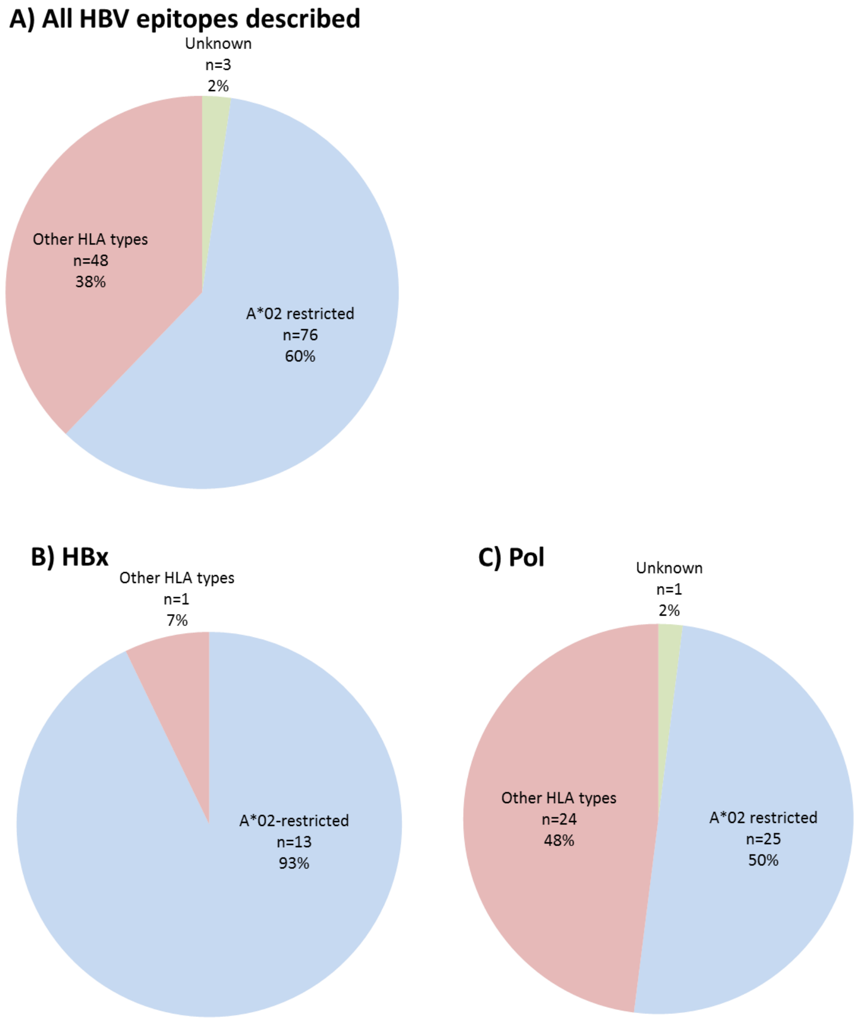

Supplementary figure 1: HLA-restriction of reported HBV-derived HLA-I epitopes. Epitopes which are thus far identified to exclusively bind the A*02 supertype were classified as A*02 restricted. All described HLA-I epitopes were classified as such derived from A) all viral proteins (n=127), B) HBx (n=14) and C) Pol (n=50).

Supplementary table 1: literature references reporting or reviewing experimental evidence for functional domains and essential amino acids of HBx.

**Essential amino acids within HBx causing loss of viral persistence upon mutation**	
Position in figure 2	Literature reference
58	[1]
61	[2]
63	[3]
68	[1]
69	[2,3]
90	[3,4]
91	[3,4]
95, 96, 98	[4]
119, 129	[1]
137	[2]
139	[1]
**Functional domains within HBx implemented in viral replication**	
Position in figure 2	Literature reference
51-72	[5,6]
88-154	[5,7]

Colors of amino acids reflect the previously calculated conservation score (methods).

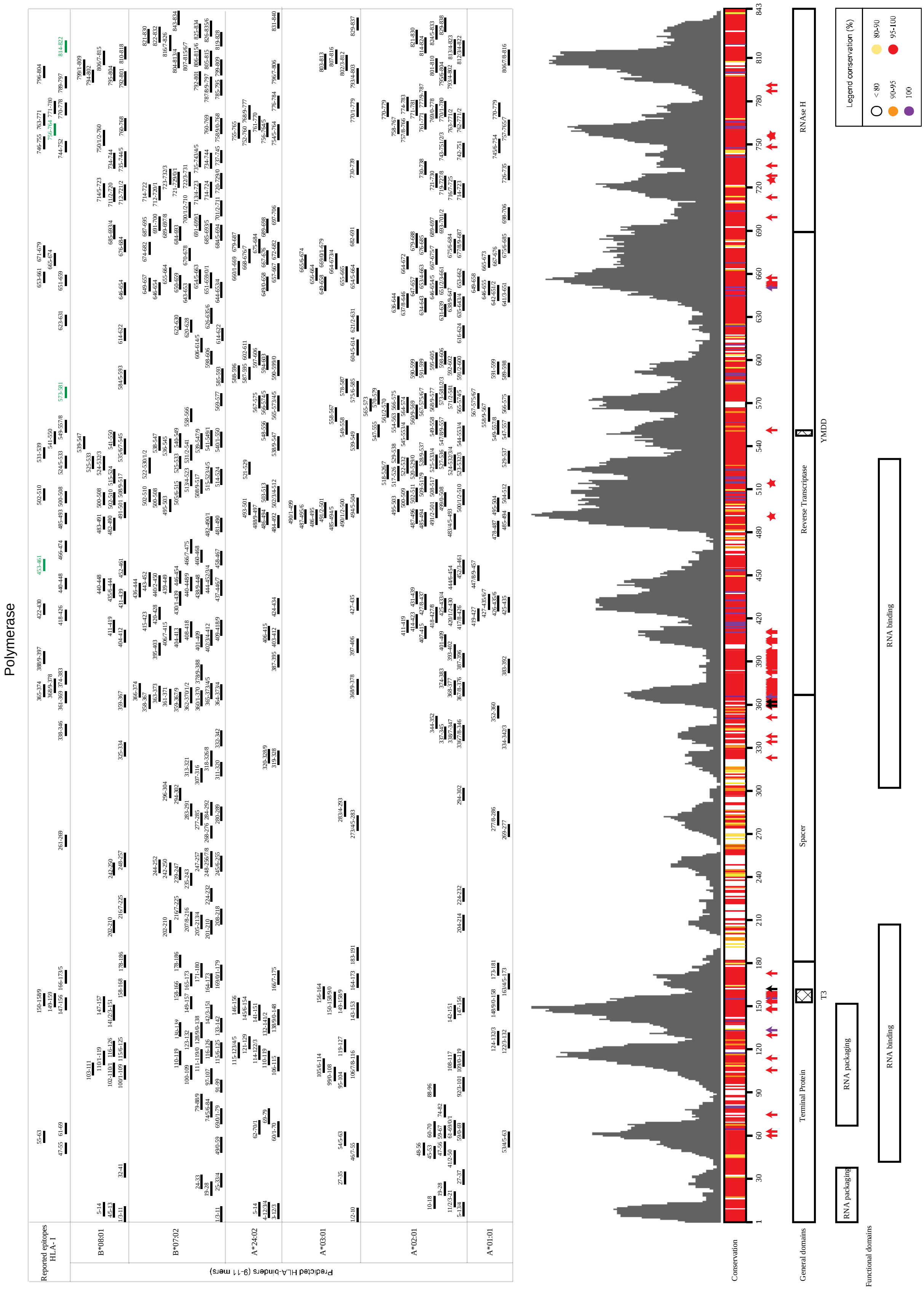

Supplementary figure 2: alignment of reported and predicted CD8+ T cell targets to protein conservation and function for full-length Polymerase. Reported epitopes obtained from the

Hepitopes database are shown on top. Responses against epitopes in green are more frequent in acute versus chronic patients ($p<0.05$). Below this, potential novel binders predicted by NetMHCpan (9-11 amino acids) are depicted for each HLA-supertype. In addition, we plotted the frequency distribution of predicted binders (8-14 amino acids in length) over the protein sequence. The conservation score (legend) of each amino acid is shown as a horizontal color coded bar diagram. Essential amino acids for which single or combined mutation leads to loss of viral persistence (≥ 50%) are indicated by arrows matching the color of the conservation score for that particular amino acid. Amino acids which are predicted to be vital in 3D confirmation are indicated with an asterisk. General domains are depicted according to previously determined nomenclature [8] in which also the T3 domain and the YMDD motif is represented. References describing the experimental evidence for essential amino acids and functional domains are listed in supplementary table 2.

Supplementary table 2: literature references reporting or reviewing experimental evidence for functional domains and essential amino acids of Pol.

**Essential amino acids within Pol causing loss of viral persistence for ≥50% upon mutation**		
Position as mentioned in reference	Position in figure 3	Literature reference
60, 63	60, 63	[9]
74	74	[10]
105	105	[11]
114, 130, 133	114, 130, 133	[9]
147	147	[10]
170, 171	147, 148	[12]
153	153	[8,9]
156	154	[8]
155	155	[8,9]
179, 180	156, 157	[8,12]
160	158	[8]
182, 160	159, 160	[8,12]
162	162	[9]
173	173	[10]
135, 146, 150, 6	323, 334, 338, 352	[13]
360	358	[8]
361	359	[8]
362, 363	360, 361	[8]
364	362	[8]
365-367	363-365	[8]
383, 368	366	[8,12]
369	367	[8]
385, 370	368	[8,12]
371-375	369-373	[8]
376, 391	374	[8,12]
377, 392	375	[8,12]
378	376	[8]
394, 379	377	[8,12]
395, 399, 400, 402	378, 382, 383, 385	[12]
388	386	[8,12]
404, 389	387	[8,12]
390-392	388-390	[8]
408, 393	391	[8,12]
409, 394	392	[8,12]
410, 395	393	[8,12]
411, 396	394	[8,12]
397	395	[8]
413, 398	396	[8,12]
399-404	397, 398, 403-406	[8]

428, 429	411, 412	[12]
146, 205	492, 515	[14]
540	551	[15]
304, 305	650, 651	[16]
652, 306	652	[17,18]
307, 308, 311	653, 654, 657	[16]
702	700	[19]
703	714	[20]
715	726	[21]
731	729	[19]
718, 725	729, 736	[15]
750	748	[19]
737	748	[15]
744, 745	755, 756	[21]
790	788	[19]
781	792	[20]
**Functional domains within Pol implemented in viral persistence**		
Position as mentioned in reference	Position in figure 3	Literature reference
1-39	1-39	[9]
67-152	67-152	[9]
42-196	42-207	[22]
291-520	302-531	[22]

Positions given in the original literature references were translated to positions within the consensus sequence by alignment (methods) and color-coded according to the calculated conservation score.

HLA-A*01:01

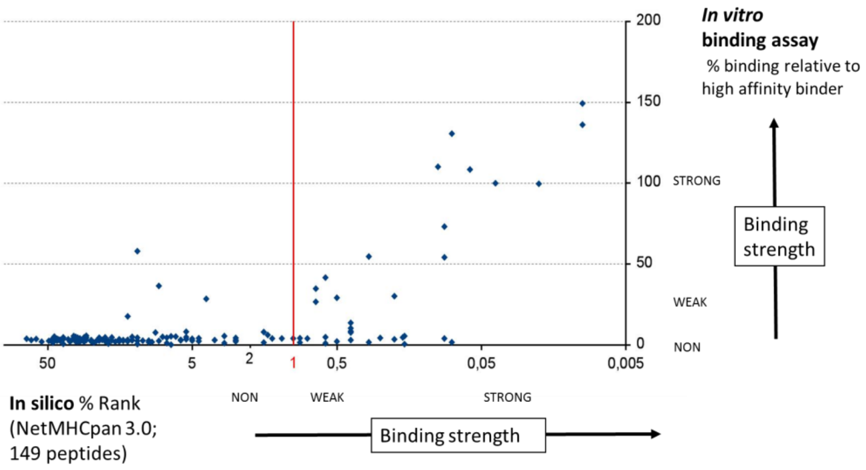

HLA-A*02:01

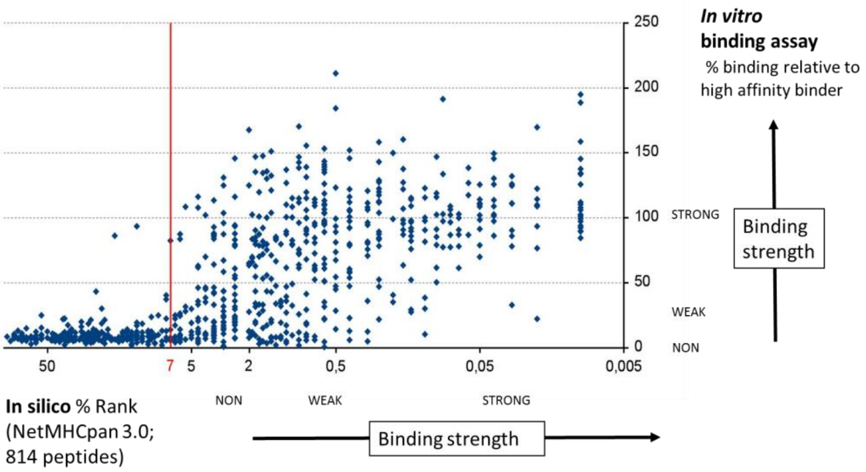

HLA-A*03:01

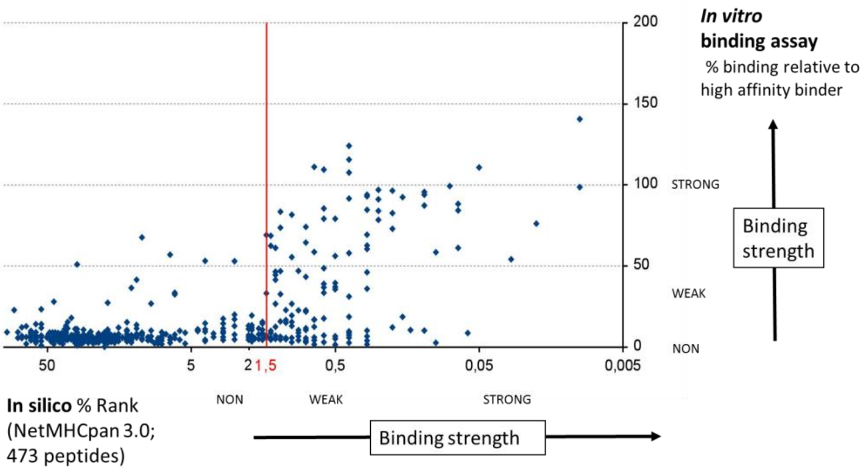

HLA-A*24:02

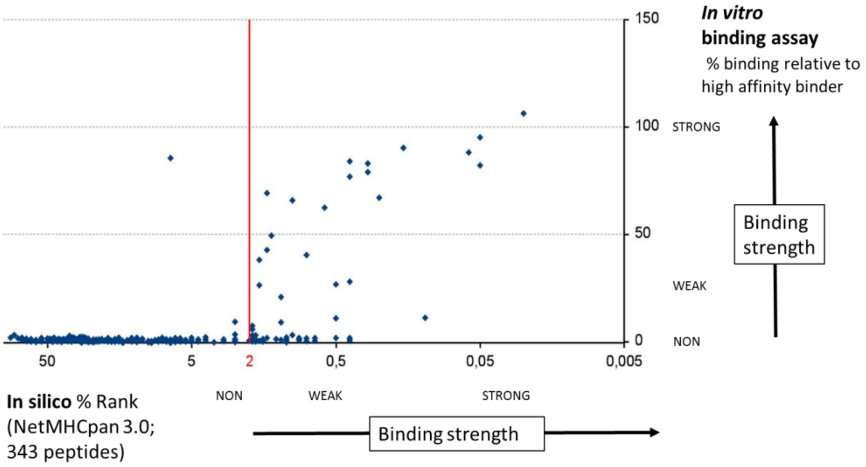

HLA-B*07:02

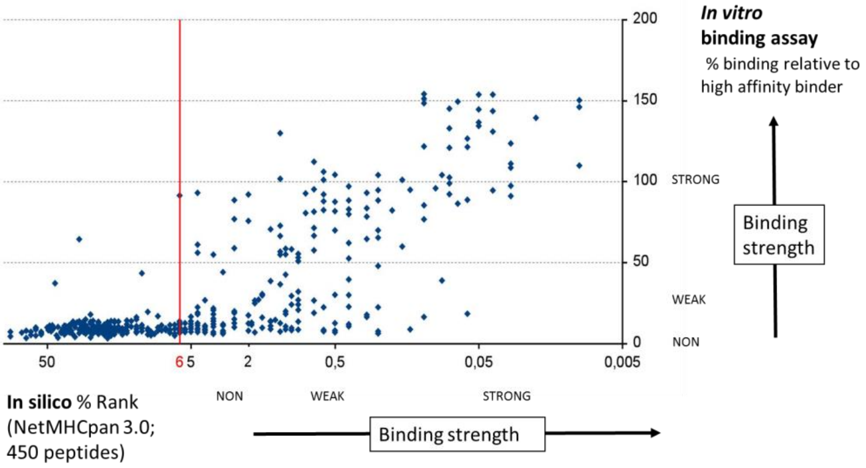

HLA-B*08:01

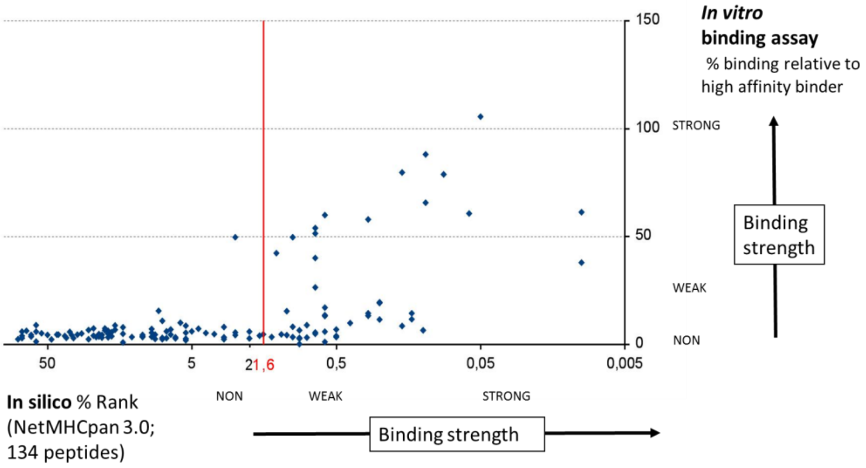

Supplementary figure 3: *in silico* predicted HLA-binding versus *in vitro* HLA-binding. *In silico*-predicted (non-)binders were tested for *in vitro* HLA-binding. Results of this assay were used to set the thresholds that discriminate predicted binders from non-binders.

Supplementary table 3: Characteristics of selected peptides to test in an *in vitro* HLA-binding assay that did not meet initial selection criteria.

Amino acid positions	Amino acid sequence	HLA type	Peptide length (amino acids)	Rank score NetMHCpan	Conservation (%)	Amino acid/ domain
x63-73	FSSAGPCALRF	A*01:01	11	1.4	99.2	yes/no
x62-73	AFSSAGPCALRF	A*24:02	12	1.7	94.2	yes/no
x110-120	AYFKDCVFKDW	A*24:02	11	1.4	59.9	yes/yes
x111-120	YFKDCVFKDW	A*24:02	10	1.2	59.9	yes/yes
x143-151	CSPAPCNFF	A*24:02	9	1.2	63.4	no/yes
x110-117	AYFKDCVF	A*24:02	8	1.0	59.9	no/yes

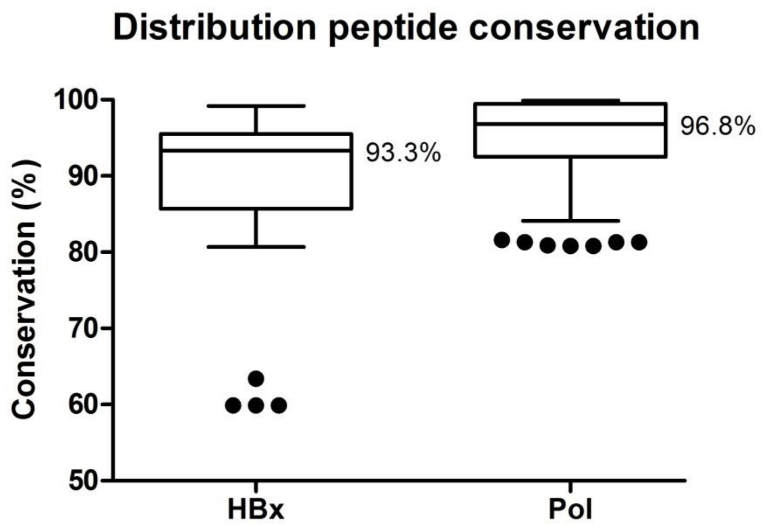

Supplementary figure 4: distribution of peptide conservation for all binders selected for *in vitro* validation. The conservation score of all peptides selected for *in vitro* verification of HLA-binding are represented in a boxplot for HBx (n=45) and Pol (n=68). The median is displayed for each set of peptides.

Supplementary table 4: control peptides used for the *in vitro* binding assay classified per HLA-type.

HLA-type	Pos ct		Neg ct	
**A*01:01**	Influenza A NP (44-52)	CTELKLSDY	RSV NP (306-314)	NPKASLLSL
**A*02:01**	CMV pp65 (495-503)	NLVPMVATV	EBV EBNA3B (416-424)	IVTDFSVIK
**A*03:01**	gp100 (614-622)	LIYRRRLMK	RSV NP (306-314)	NPKASLLSL
**A*11:01**	EBV EBNA3B (416-424)	IVTDFSVIK	RSV NP (306-314)	NPKASLLSL
**A*24:02**	GPR143 (126-134)	LYSACFWWL	CMV pp65 (495-503)	NLVPMVATV
**B*07:02**	RSV NP (306-314)	NPKASLLSL	gp100 (614-622)	LIYRRRLMK
**B*08:01**	Influenza A NP (380-388)	ELRSRYWAI	CMV pp65 (495-503)	NLVPMVATV

References

1. Sitterlin D, Lee T, Prigent S, Tiollais P, Butel JS, Transy C. 1997. Interaction of the UV-damaged DNA-binding protein with hepatitis B virus X protein is conserved among mammalian hepadnaviruses and restricted to transactivation-proficient X-insertion mutants. J Virol. 71:6194–9.

2. Kumar V, Jayasuryan N, Kumar R. 1996. A truncated mutant (residues 58-140) of the hepatitis B virus X protein retains transactivation function. Proc Natl Acad Sci U S A. 93:5647–52.

3. Becker SA, Lee TH, Butel JS, Slagle BL. 1998. Hepatitis B virus X protein interferes with cellular DNA repair. J Virol. 72:266–72.

4. Lin-Marq N, Bontron S, Leupin O, Strubin M. 2001. Hepatitis B virus X protein interferes with cell viability through interaction with the p127-kDa UV-damaged DNA-binding protein. Virology. 287:266–74.

5. Tang H, Delgermaa L, Huang F, Oishi N, Liu L, He F. 2005. The Transcriptional Transactivation Function of HBx Protein Is Important for Its Augmentation Role in Hepatitis B Virus Replication. J Virol. 79:5548–56.

6. Murakami S, Cheong J, Kaneko S. 1994. Human hepatitis virus X gene encodes a regulatory domain that represses transactivation of X protein. J Biol Chem. 269:15118–23.

7. Gong D, Chen E, Huang F, Leng X, Cheng X, Tang H. 2013. Role and functional domain of hepatitis B virus X protein in regulating HBV transcription and replication in vitro and in vivo. Viruses. 5:1261–71.

8. Cao F, Jones S, Li W, Cheng X, Hu Y, Hu J, Tavis JE. 2014. Sequences in the terminal protein and reverse transcriptase domains of the hepatitis B virus polymerase contribute to RNA binding and encapsidation. J Viral Hepat. 21:882–93.

9. Clark DN, Flanagan JM, Hu J. 2017. Mapping of Functional Subdomains in the Terminal Protein Domain of Hepatitis B virus Polymerase. J Virol. 91:e01785-16.

10. Shin Y, Ko C, Ryu W. 2011. Hydrophobic residues of terminal protein domain of hepatitis B virus polymerase contribute to distinct steps in viral genome replication. FEBS Lett. 585:3964–8.

11. Shin YC, Park S, Ryu W. 2011. A conserved arginine residue in the terminal protein domain of hepatitis B virus polymerase is critical for RNA pre-genome encapsidation. J Gen Virol. 92:1809–16.

12. Badtke MP, Khan I, Cao F, Hu J, Tavis JE. 2009. An interdomain RNA binding site on the hepadnaviral polymerase that is essential for reverse transcription. Virology. 390:130–8.

13. Kim S, Lee J, Ryu W. 2009. Four Conserved Cysteine Residues of the Hepatitis B Virus Polymerase Are Critical for RNA Pregenome Encapsidation. J Virol. 83:8032–40.

14. Xu X, Thai H, Kitrinos KM, Xia G, Gaggar A, Paulson M, Ganova-Raeva L, Khudyakov Y, Lara J. 2016. Modeling the functional state of the reverse transcriptase of hepatitis B virus and its application to probing drug-protein interaction. BMC Bioinformatics. 17 Suppl 8:280.

15. Radziwill G, Tucker W, Schaller H. 1990. Mutational analysis of the hepatitis B virus P gene product: domain structure and RNase H activity. J Virol. 64:613–20.

16. Wang Y, Xu X, Luo C, Ma Z, Jiang H, Ding J, Wen Y. 2007. A Putative New Domain Target for Anti-Hepatitis B Virus : Residues Flanking Hepatitis B Virus Reverse Transcriptase Residue 306 (rtP306). J Med Virol. 79:676–82.

17. Lin X, Yuan Z, Wu L, Ding J, Lin XU, Wu LI. 2001. A Single Amino Acid in the Reverse Transcriptase Domain of Hepatitis B Virus Affects Virus Replication Efficiency. J Virol. 75:11827–33.

18. Wang Y, Xu X, Luo C, Ma Z, Jiang H, Ding J, Wen Y. 2007. Mutational analysis revealed that conservation of hepatitis B virus reverse transcriptase residue 306 (rtP306) is crucial for encapsidation of pregenomic RNA. FEBS Lett. 581:558–64.

19. Tavis JE, Cheng X, Hu Y, Totten M, Cao F, Michailidis E, Aurora R, Meyers MJ, Jacobsen EJ, Parniak MA, Sarafianos SG. 2013. The Hepatitis B Virus Ribonuclease H Is Sensitive to Inhibitors of the Human Immunodeficiency Virus Ribonuclease H and Integrase Enzymes. PLoS Pathog. 9:e1003125.

20. Ko C, Shin Y, Park W, Kim S, Kim J, Ryu W. 2014. Residues Arg703, Asp777, and Arg781 of the RNase H Domain of Hepatitis B Virus Polymerase Are Critical for Viral DNA Synthesis. J Virol. 88:154–63.

21. Potenza N, Salvatore V, Raimondo D, Falanga D, Nobile V, Peterson DL, Russo A. 2007. Optimized expression from a synthetic gene of an untagged RNase H domain of human hepatitis B virus polymerase which is enzymatically active. Protein Expr Purif. 55:93–9.

22. Hu J, Boyer M. 2006. Hepatitis B Virus Reverse Transcriptase and ε RNA Sequences Required for Specific Interaction In Vitro. J Virol. 80:2141–50.
